# Supplementary figures and images for: 2 Hydroxybutyric Acid-Producing Bacteria in Gut Microbiome and Fusobacterium nucleatum Regulates 2 Hydroxybutyric Acid Level In Vivo
Source: Metabolites. 2023 Mar 20;13(3):451. doi: 10.3390/metabo13030451 (PMC10059959; doi:10.3390/metabo13030451)

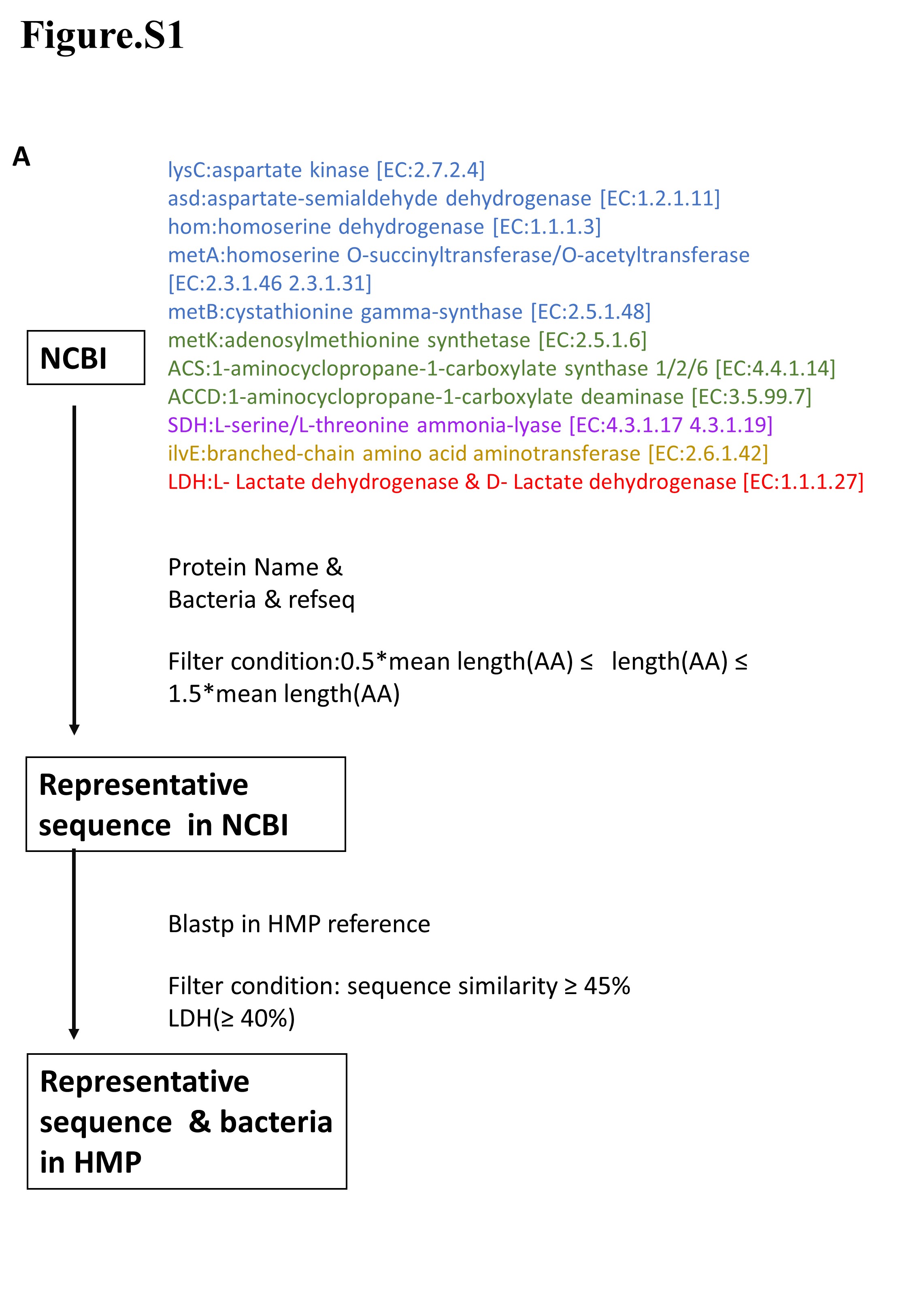

Supplement: Supplementary file 1 [file metabolites-13-00451-s001.zip › Figure S1.jpg]

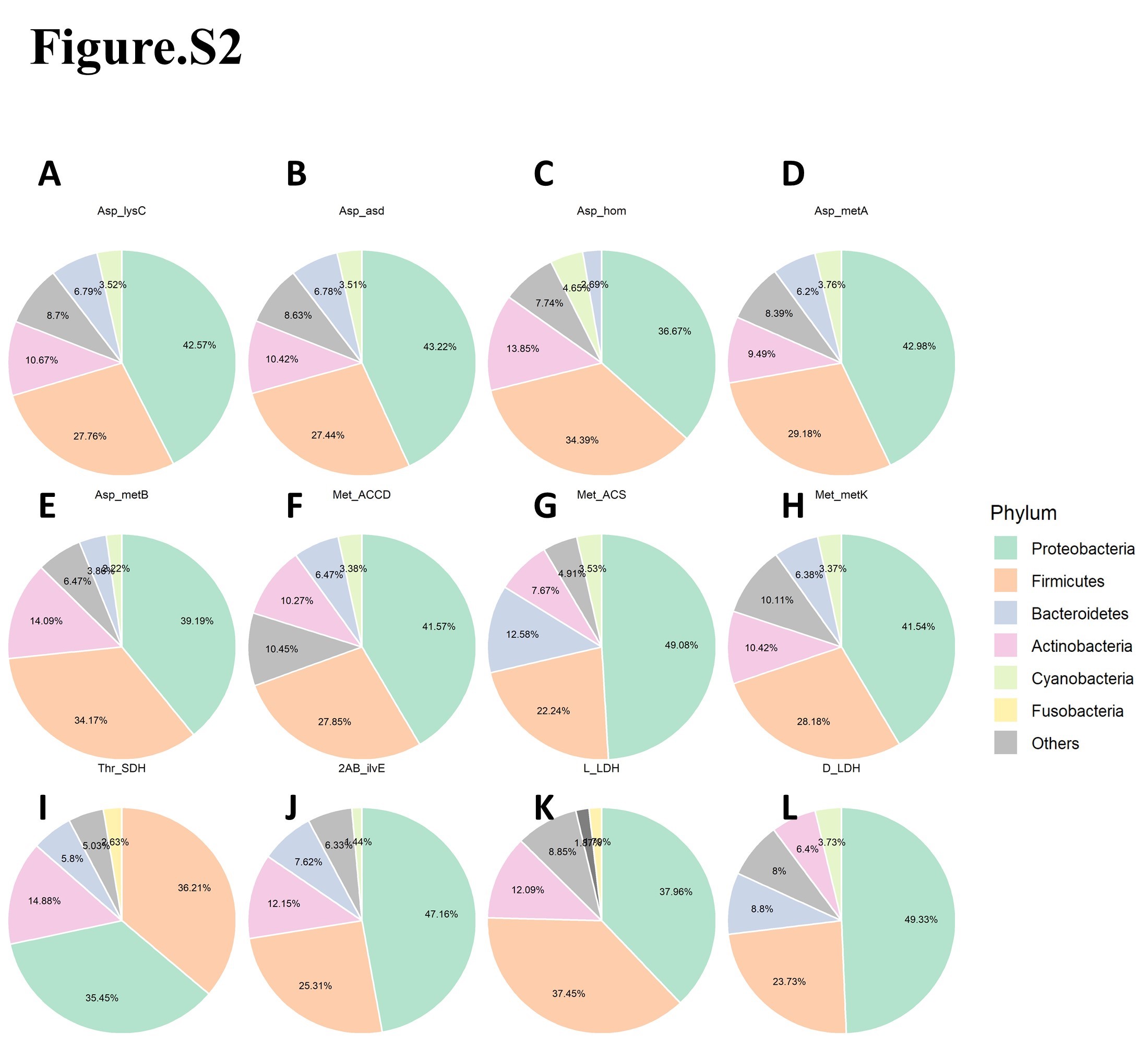

Supplement: Supplementary file 1 [file metabolites-13-00451-s001.zip › Figure S2.jpg]
